# Comorbidities, Socioeconomic Status, and Colorectal Cancer Diagnostic Route

**DOI:** 10.1001/jamanetworkopen.2025.8867

**Published:** 2025-05-06

**Authors:** Flavia Pennisi, Carlotta Buzzoni, Antonio Giampiero Russo, Federico Gervasi, Mario Braga, Cristina Renzi

**Affiliations:** 1 School of Medicine, University Vita-Salute San Raffaele, Milan, Italy; 2 PhD National Programme in One Health Approaches to Infectious Diseases and Life Science Research, Department of Public Health, Experimental and Forensic Medicine, University of Pavia, Pavia, Italy; 3 Epidemiology Unit, Agency for Health Protection of Milan, Milan, Italy; 4 Department of Environmental and Preventive Sciences, University of Ferrara, Ferrara, Italy; 5 Research Department of Behavioural Science and Health, University College London, London, United Kingdom

## Abstract

**Importance:**

Reducing emergency cancer diagnoses is a public health priority, as they are associated with worse outcomes. Preexisting chronic conditions can influence screening participation and emergency cancer diagnosis; however, evidence is mixed, and data from Southern Europe are scant.

**Objective:**

To examine variations in the likelihood of colorectal cancer (CRC) diagnosis following an emergency presentation (EP) or screening by patient comorbidity status and socioeconomic characteristics and to investigate the association of patient characteristics, diagnostic route, and comorbidity status with short-term CRC mortality.

**Design, Setting, and Participants:**

This population-based cohort study used linked cancer registry data and administrative health data from the Agency for Health Protection of Milan, Italy, for CRC cases diagnosed between July 1, 2014, and December 31, 2020, in the provinces of Milan and Lodi, Italy. Data were analyzed from January 1 to October 1, 2024.

**Exposures:**

Comorbidity status (number of comorbidities, specific preexisting comorbidities) and socioeconomic characteristics (including age, sex, and deprivation index).

**Main Outcomes and Measures:**

The primary outcomes were routes to cancer diagnosis (screening, emergency presentation, or inpatient or outpatient visits), cancer stage at diagnosis, and short-term mortality (30 days and 1 year). Multivariable and multinomial logistic regression models were used to estimate odds ratios (ORs) adjusted for socioeconomic and comorbidity factors.

**Results:**

Among 14 457 patients, 10 750 (74.4%) had colon cancer and 3707 (25.6%) had rectal cancer. The route to diagnosis was reconstructed for 10 514 patients with colon cancer (97.8%; median age, 73.1 years [IQR, 66-82 years]; 5563 [52.9%] male) and 3635 with rectal cancer (98.1%; median age, 70.3 years [IQR, 62-80 years]; 2079 [57.2%] male). Of those, 4697 patients with colon cancer (44.6%) and 2094 with rectal cancer (57.6%) had comorbidities, emergency diagnosis occurred in 3738 colon (35.6%) and 823 rectal (22.6%) cancer cases, and diagnosis while screening occurred in 881 colon (8.4%) and 347 rectal (9.5%) cancer cases. Emergency diagnosis was associated with having cerebrovascular (adjusted OR [AOR], 1.50; 95% CI, 1.23-1.82) and neurological (AOR, 1.67; 95% CI, 1.33-2.09) diseases or having 3 or more comorbidities compared with having none (AOR, 1.78; 95% CI, 1.47-2.16) among patients with colon cancer. Having 3 or more vs no comorbidities was associated with lower odds of screening-detected colon cancer (AOR, 0.64; 95% CI, 0.45-0.91). Higher 30-day (AOR, 4.84; 95% CI, 2.81-8.33) and 1-year (AOR, 2.77; 95% CI, 2.17- 3.53) mortality was associated with emergency presentation. The COVID-19 period was associated with higher odds of emergency diagnoses compared with the prepandemic period (AOR, 1.32; 95% CI, 1.15-1.52).

**Conclusions and Relevance:**

In this cohort study of patients with CRC in Italy, emergency diagnosis occurred for more than 1 in 3 patients with colon cancer. Having 3 or more comorbidities was associated with a lower likelihood of screening detection and higher odds of emergency diagnosis. Tailored interventions are needed to facilitate screening, to reduce emergency cancer diagnoses, and to improve outcomes for patients with chronic conditions.

## Key Points

**Question:**

Are comorbidities and socioeconomic characteristics associated with the diagnostic route and health outcomes in patients with colorectal cancer (CRC)?

**Findings:**

In this cohort study of 14 457 patients with CRC, 36% with colon cancer and 23% with rectal cancer were diagnosed through emergency presentation, with higher short-term mortality vs diagnosis by screening or during inpatient or outpatient visits. Presence of 3 or more comorbidities vs none was associated with lower screening detection and higher odds of emergency diagnosis.

**Meaning:**

These findings highlight the need for targeted interventions and use of comorbidity status to prioritize symptomatic patients for urgent CRC investigations.

## Teaser

This cohort study examines incidence and mortality outcomes after colorectal cancer diagnosis through emergency presentation, screening, or inpatient or outpatient visit by patient comorbidity status and socioeconomic characteristics.

## Introduction

Reducing emergency colorectal cancer (CRC) diagnosis is a public health priority,^1^ as emergency diagnosis is associated with worse survival compared with other diagnostic routes independently of cancer stage at diagnosis.^2,3^ Despite improvements in screening and early diagnosis, the proportion of colon cancer diagnoses following an emergency presentation ranges from 23% to 37% in Northern Europe, Australia, and Canada.^1^ Data on CRC diagnoses following an emergency presentation in Southern Europe are scant. Italy, having among the highest cancer survival rates in Europe^4^ and with a national CRC screening program since 2005,^5^ can provide an opportunity for bridging this evidence gap on diagnostic routes and related risk factors. In Lombardy, Italy, CRC screening was offered to individuals aged 50 to 69 years from 2014 to 2017 and was extended to age 74 years from 2017 onward.

Socioeconomic characteristics, geographic differences,^6^ and patient clinical characteristics, including preexisting chronic conditions (ie, comorbidities), may be associated with the diagnostic route and health outcomes of cancer. Recent data indicate that 23.7 million Italians have at least 1 chronic disease, which may affect cancer incidence, diagnostic route, and/or outcomes.^7^ Comorbidities can influence screening participation, help-seeking for new or evolving symptoms,^8^ and clinicians’ decision-making regarding differential diagnosis and cancer investigation strategies.^9,10^ According to UK data, many patients with comorbidities repeatedly present to a physician with cancer-related symptoms in the 2 years before being diagnosed with cancer through emergency presentation.^11^ Understanding the role of comorbidities in cancer diagnosis can help improve care and health outcomes for the rising number of people with chronic conditions in Western populations.

The COVID-19 pandemic led to cancer diagnostic delays due to the cancellation of surgical procedures and the suspension of screening programs.^12,13,14^ In Italy, a decrease of more than 30% in CRC screening was observed between March 2020 and May 2021.^15^ However, there are few studies based on Italian cancer data assessing the association between the pandemic and diagnostic routes or outcomes.^16,17^ Lombardy is of particular interest, having been the first region in Europe affected by the pandemic in February 2020.^18,19^

The primary objective of our study was to examine variations in CRC diagnostic routes by comorbidity status and socioeconomic characteristics among patients with CRC in northern Italy during the prepandemic period (July 2014 to February 2020). Additionally, we investigated the association of diagnostic routes, comorbidity status, and patient characteristics with short-term mortality. As a secondary objective, we conducted an exploratory analysis to assess whether the early phase of the COVID-19 pandemic was associated with variations in diagnostic routes.

## Methods

### Study Design and Study Population

We conducted a retrospective, population-based cohort study using Milan Cancer Registry data linked to administrative health databases (AHDs) from the Agency for Health Protection (ATS) of Milan, part of the Regional Health Service of the Lombardy region in northern Italy. The study followed the Strengthening the Reporting of Observational Studies in Epidemiology (STROBE) reporting guideline. Approval for the study was granted by the Comitato Etico Territoriale Lombardia 3 ethics committee. The personal and health data required for the study, obtained from the Cancer Registry of ATS Milan and its nonpermanently linked health databases, were processed based on the fulfilment of a legal obligation of the data controller (ATS of Milan) and the performance of a task carried out in the public interest (European Union Regulation 2016/679). Based on this legal framework, the informed consent of each participant was not required. No identifiable data were shared for this project.

The study included the provinces of Milan and Lodi (193 municipalities), with a population of about 3.46 million people in 2017. Deterministic record linkages were performed using anonymized individual codes of health system beneficiaries. CRC cases diagnosed between July 1, 2014, and December 31, 2020, among residents in the area of the ATS of Milan were included (*International Classification of Diseases for Oncology, Third Edition* topographic codes C18 through C20 in association with behavior code 3). For the main analyses, we included CRC diagnosed during the prepandemic period (July 2014 to February 2020). CRC diagnoses during the initial pandemic period (March to December 2020) were only included in the secondary analyses to evaluate whether the pandemic was associated with variations in diagnostic routes.

### Data Sources and Variable Definitions

Sex, subsite, age, calendar year, and cancer stage at diagnosis were selected from the Milan Cancer Registry, which is part of the Italian Association of Cancer Registries and the International Association of Cancer Registries.^20^ Patients’ socioeconomic level was evaluated using the deprivation index calculated from Italian census data^21^ at the census section level, adopting the Rosano revised version of the Caranci Index.^22,23,24^ The index considers 5 socioeconomic traits and is divided into 5 levels corresponding to the quintiles of its distribution (quintile 1 indicates the least deprived census sections).

Preexisting comorbidities among study patients were identified according to the criteria and codes established by the 2017 Lombardy region’s deliberation No. X/6164, linking health records up to 6 months before diagnosis. Five non–mutually exclusive chronic conditions were considered: cardiovascular diseases (CVDs); type 2 diabetes (T2D); and neurological, genitourinary, and cerebrovascular diseases. The total number of conditions was also calculated for each patient.

### Definition of Routes to Diagnosis

A route to diagnosis is defined as the sequence of interactions between the patient and the health care system leading to a cancer diagnosis.^25^ As there is no synthetic information about route to diagnosis in the Italian AHD, we developed an algorithm to identify the most likely route based on each patient’s contacts with the health care system in the 6-month period before the CRC diagnosis.

Diagnostic and therapeutic procedures provided to study patients (coded per the *International Classification of Diseases, 9th Revision—Clinical Modification* of the World Health Organization), with associated dates of service, were selected from 3 data sources: hospital discharge records, the outpatient database, and the emergency department discharge database (which provided emergency presentations). Three mutually exclusive routes to diagnosis were identified^1^: screening,^2^ emergency presentation, and inpatient or outpatient visits.^3^ For each patient, the route was defined based on the temporal proximity between the date of diagnosis and the dates of service of the identified diagnostic and therapeutic procedures.

**Figure 1.**
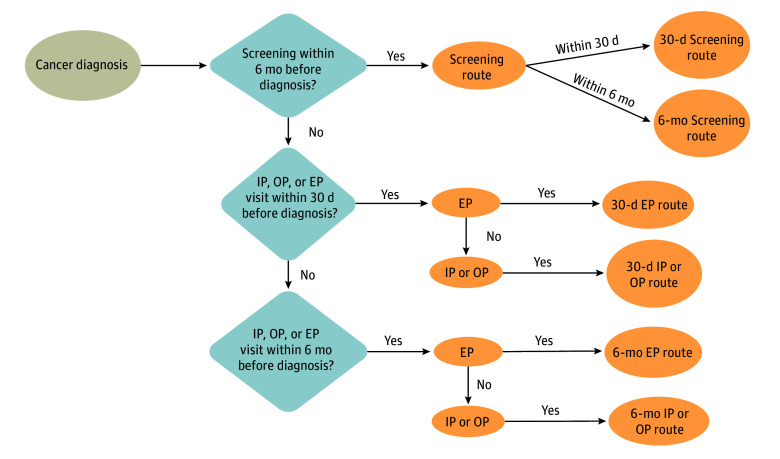
Definition of Route to Colorectal Cancer Diagnosis EP indicates emergency presentation; IP, inpatient; OP, outpatient.

First, contacts with the health care system in the 30 days before diagnosis were considered, defining a reasonable period to enable a histologically verified diagnosis, a time frame commonly adopted in the field of health care services evaluation. Only in the absence of a contact within 30 days were contacts in the 6 months before diagnosis taken into account. The British route to diagnosis used a similar approach.^25^ For patients with multiple contacts within each period, by the definition of route to diagnosis, priority was given as follows: (1) screening, (2) emergency presentation, and (3) inpatient or outpatient visits (Figure 1).

### Statistical Analysis

Descriptive statistics of the study population, including recorded comorbidities, diagnostic route, and cancer stage at diagnosis, were assessed using χ^2^ tests for comparisons. The associations between the variables are shown in the eFigure in Supplement 1.

Focusing on individuals diagnosed with cancer in the prepandemic period (July 2014 to February 2020), 5 multivariable and multinomial logistic regression models were developed separately for colon and rectal cancers. Model A was a multivariable logistic regression model fitted to estimate the adjusted odds ratio (AOR) of cancer diagnosis following emergency presentation vs nonemergency presentation, including as explanatory variables sex, age, deprivation index, marital status, and specific comorbidities. Model B was a multivariable logistic regression model with the same outcome as model A but included as explanatory variables sex, age, educational level, marital status, and comorbidity count (0, 1, 2, or ≥3). Model C was a multivariable logistic regression model with the same outcome as models A and B but included as explanatory variables sex, age, deprivation index, marital status, comorbidity count, and cancer stage at diagnosis. Model D was a multinomial logistic regression model estimating the AOR of cancer diagnosis following emergency presentation and screening vs inpatient or outpatient visits, including as explanatory variables sex, age, deprivation index, marital status, and comorbidity count. Model E was a multivariable logistic regression model to estimate the AOR of short-term mortality at 30 days and 1 year, including as explanatory variables diagnostic pathway, socioeconomic characteristics, comorbidity count, and cancer stage at diagnosis. Model E was also repeated excluding cancer stage.

Additionally, for our secondary objective, we estimated the association between the initial pandemic period and route to diagnosis, including all patients with cancer diagnosed between July 2014 and December 2020. Model D was used for this analysis, with year of diagnosis (pandemic vs prepandemic) as an additional explanatory variable. Multimorbidity was defined as the cooccurrence of at least 2 chronic conditions in the same individual.^26,27^

We used SAS software, version 9.4 (SAS Institute Inc) for data management and all statistical analyses. Data were analyzed from January 1 to October 31, 2024. Two-sided *P* <.05 was considered significant.

## Results

### Participant Characteristics

The study included 14 457 patients: 10 750 (74.4%) with colon cancer and 3707 (25.6%) with rectal cancer. The route to diagnosis was reconstructed for 10 514 patients with colon cancer (97.8%; median age, 73.1 years [IQR, 66-82 years]; 4951 [47.1%] female; 5563 [52.9%] male) and 3635 with rectal cancer (98.1%; median age, 70.3 years [IQR, 62-80 years]; 1556 [42.8%] female; 2079 [57.2%] male). The route to diagnosis was screening in 881 cases of colon cancer (8.4%) and 347 cases of rectal cancer (9.5%); 3738 colon cancer cases (35.6%) were diagnosed through emergency presentation and 5895 (22.6%) through inpatient or outpatient visits, and 823 rectal cancer cases (22.6%) were diagnosed through emergency presentation and 2466 (67.8%) through inpatient or outpatient visits. Specifically, 9538 patients with colon cancer (90.7%) had their last contact with the health care system within 30 days before diagnosis, of whom 3553 (37.3%) were diagnosed through emergency presentation and 585 (6.1%) through screening. For rectal cancer, 3314 patients (91.2%) entered the diagnostic route within 30 days, of whom 763 (23.0%) were diagnosed through emergency presentation and 237 (7.2%) through screening (eTable 1 in Supplement 1). A small number of patients (10 [0.7%]) underwent screening outside the target age range, most of whom were aged 49 years (7 [70.0%]).

Overall, among patients with colon and rectal cancer, 4697 (44.6%) and 2094 (57.6%), respectively, had 1 or more preexisting comorbidity, most frequently hypertension (4864 [53.7%] colon and 1552 [42.7%] rectal cancer), CVD (2477 [23.6%] colon and 661 [18.2%] rectal cancer), and T2D (1624 [15.4%] colon and 539 [14.8%] rectal cancer) (Table 1). The probability of emergency presentation as the diagnostic route was significantly higher in younger and older age groups (224 of 474 patients younger than 50 years [47.3%] and 234 of 438 older than 90 years [53.4%]), those with higher levels of deprivation, those with lower educational level, those who were single or widowed, and those with higher comorbidity burden (Table 1).

**Table 1.** Patient Characteristics and Diagnostic Routes^a^

Characteristic	Colon cancer					Rectal cancer				
	Total (n = 10 514)	EP (n = 3738)	Screening (n = 881)	IP or OP visit (n = 5895)	*P* value^b^	Total (n = 3635	EP (n = 823)	Screening (n = 347)	IP or OP visit (n = 2466)	*P* value^b^
Age, y										
<50	474 (4.5)	224 (47.3)	6 (1.3)	244 (51.5)	<.001	221 (6.1)	35 (15.8)	3 (1.4)	183 (82.8)	<.001
50-59	952 (9.1)	271 (28.4)	239 (25.1)	442 (46.4)		522 (14.4)	78 (14.9)	112 (21.4)	332 (63.7)	
60-69	2008 (19.1)	508 (25.3)	464 (23.1)	1036 (51.6)		831 (22.9)	127 (15.3)	176 (21.3)	528 (63.5)	
70-79	3501 (33.3)	1162 (33.2)	171 (4.9)	2168 (61.9)		1138 (31.3)	255 (22.4)	55 (4.8)	828 (72.8)	
80-89	3141 (29.9)	1339 (42.6)	1 (0.03)	1801 (57.3)		808 (22.2)	263 (32.5)	0	545 (67.5)	
≥90	438 (4.2)	234 (53.4)	0	204 (46.6)		115 (3.2)	65 (56.5)	0	50 (43.5)	
Sex										
Female	4951 (47.1)	1719 (34.7)	443 (9.0)	2789 (56.3)	.06	1556 (42.8)	366 (23.5)	177 (11.4)	1013 (65.1)	<.001
Male	5563 (52.9)	2019 (36.3)	438 (7.9)	3106 (55.8)		2079 (57.2)	457 (22.0)	169 (8.1)	1453 (69.9)	
Deprivation index quintile^c^										
1	2214 (21.6)	711 (32.1)	186 (8.4)	1317 (59.5)	<.001	749 (21.2)	141 (18.8)	53 (7.1)	555 (74.1)	<.001
2	1815 (17.7)	614 (33.8)	151 (8.3)	1050 (57.9)		643 (18.2)	125 (19.4)	79 (12.3)	439 (68.3)	
3	1709 (16.6)	591 (34.6)	159 (9.3)	959 (56.1)		631 (17.8)	146 (23.1)	74 (11.7)	411 (65.1)	
4	1850 (18.0)	678 (36.7)	154 (8.3)	1018 (55.0)		596 (16.8)	163 (27.4)	58 (9.7)	375 (62.9)	
5	2687 (26.2)	1061 (39.5)	209 (7.8)	1417 (52.7)		923 (26.1)	225 (24.4)	76 (8.2)	622 (67.4)	
Missing	239 (2.3)	NA	NA	NA		93 (2.5)	NA	NA	NA	
Marital status										
Single	1081 (10.4)	431 (39.8)	87 (8.0)	563 (52.1)	<.001	419 (11.7)	87 (20.8)	32 (7.6)	300 (71.6)	<.001
Married	6272 (60.1)	2027 (32.3)	643 (10.3)	3602 (57.4)		2277 (63.3)	465 (20.4)	246 (10.8)	1566 (68.8)	
Widowed	2336 (22.4)	1010 (43.2)	57 (2.4)	1269 (54.3)		606 (16.8)	205 (33.8)	17 (2.8)	384 (63.4)	
Divorced	352 (3.4)	116 (33.0)	26 (7.4)	210 (60.0)		151 (4.2)	38 (25.2)	19 (12.6)	94 (62.3)	
Missing	500 (4.7)	NA	NA	NA		37 (1.0)	NA	NA	NA	
Educational level										
None or primary school	2772 (26.4)	1164 (42.0)	103 (3.7)	1505 (54.2)	<.001	857 (23.6)	282 (32.9)	28 (3.3)	547 (63.8)	<.001
Secondary school	3066 (29.2)	1123 (36.6)	252 (8.2)	1691 (55.2)		1038 (28.6)	246 (23.7)	97 (9.3)	695 (66.9)	
Diploma, degree, or PhD	3516 (33.4)	1078 (30.7)	366 (10.4)	2072 (58.9)		1325 (36.4)	218 (16.5)	158 (11.9)	949 (71.6)	
Missing	1160 (11.0)	NA	NA	NA		415 (11.4)	NA	NA	NA	
Comorbidities, No.										
0	5817 (55.3)	1924 (33.1)	650 (11.2)	3243 (55.8)	<.001	1541 (42.4)	289 (18.8)	177 (11.5)	1075 (69.8)	<.001
1	2874 (27.3)	1043 (36.3)	159 (5.5)	1672 (58.2)		1085 (29.9)	228 (21.0)	109 (10.1)	748 (68.9)	
2	1250 (11.9)	488 (39.0)	57 (4.6)	705 (56.4)		613 (16.9)	169 (27.6)	40 (6.5)	404 (65.9)	
3	398 (3.8)	197 (49.5)	12 (3.0)	189 (47.5)		268 (7.4)	88 (32.8)	13 (4.9)	167 (62.3)	
≥4	175 (1.7)	86 (49.1)	3 (1.7)	86 (49.1)		128 (3.5)	49 (38.3)	7 (5.5)	72 (56.3)	
Specific comorbidities										
CVD	2477 (23.6)	1046 (42.2)	78 (3.2)	1353 (54.6)	<.001	661 (18.2)	240 (36.3)	20 (3.0)	401 (60.7)	<.001
Cerebrovascular diseases	546 (5.2)	280 (51.3)	14 (2.6)	252 (46.2)	<.001	129 (3.6)	50 (38.5)	2 (1.5)	77 (60.0)	<.001
Type 2 diabetes	1624 (15.4)	617 (38.0)	88 (5.4)	919 (56.6)	<.001	539 (14.8)	142 (26.4)	41 (7.6)	356 (66.1)	.04
Complicated type 2 diabetes	192 (1.8)	94 (49.0)	5 (2.6)	93 (48.4)	<.001	46 (1.3)	21 (45.7)	1 (2.17)	24 (52.2)	<.001
Endocrine and metabolic diseases	467 (4.4)	146 (31.3)	50 (10.7)	271 (58.0)	.049	130 (3.6)	24 (18.5)	23 (17.7)	83 (63.9)	.005
Neurological diseases	390 (3.7)	199 (51.0)	10 (2.6)	181 (46.4)	<.001	96 (2.6)	35 (36.5)	7 (7.3)	54 (56.3)	.005
Hypertension	4864 (53.7)	1838 (37.8)	274 (5.6)	2752 (56.6)	<.001	1552 (42.7)	406 (26.1)	121 (7.8)	1025 (66.0)	<.001
Genitourinary diseases	401 (3.8)	176 (43.9)	6 (1.5)	219 (54.6)	<.001	107 (2.9)	37 (34.6)	7 (6.5)	63 (58.9)	.01

Abbreviations: CVD, cardiovascular disease; EP, emergency presentation; IP, inpatient; NA, not applicable; OP, outpatient.

^a^ Data are presented as number (percentage) of patients among the row total for each cancer type unless otherwise indicated.

^b^ *P* values refer to comparisons between the different diagnostic routes (EP, screening, and IP or OP visit).

^c^ Quintile 1 indicates least deprived.

When considering specific chronic diseases, the probability of an emergency presentation diagnostic route vs diagnosis through screening or an inpatient or outpatient visit was higher among people with cerebrovascular or neurological diseases and complicated T2D. The probability of screening route was highest in people aged 50 to 69 years, those with higher educational levels, married individuals, and those with no comorbidity. Patients diagnosed through emergency presentation had a higher frequency of stage 4 cancer at diagnosis and greater short-term mortality compared with the other routes of diagnosis (eTable 2 in Supplement 1).

### Multivariable Analyses for the Likelihood of Emergency Presentation by Patient Characteristics and Comorbidity Status

Model A (Table 2) showed that for colon cancer, emergency presentation was significantly associated with younger and older age groups (<50 years and ≥70 years); higher deprivation index; marital status of single or widowed; and cardiovascular (AOR, 1.26; 95% CI, 1.13-1.41), neurological (AOR, 1.67; 95% CI, 1.33-2.09), and cerebrovascular (AOR, 1.50; 95% CI, 1.23-1.82) diseases. The presence of at least 1 chronic condition was significantly associated with higher odds of emergency presentation diagnosis compared with no chronic conditions (1 comorbidity: AOR, 1.12 [95% CI, 1.01-1.25]; 2 comorbidities: AOR, 1.27 [95% CI, 1.10-1.46]; ≥3 comorbidities: AOR, 1.78 [95% CI, 1.47-2.16]) (model B) (eTable 3 in Supplement 1). Similarly, a higher likelihood of emergency presentation diagnosis associated with an increasing number of comorbidities was observed when including the cancer stage in the model (model C) (eTable 4 in Supplement 1). A sensitivity analysis restricted to emergency presentation occurring within 30 days before diagnosis yielded similar results.

**Table 2.** Association of Patient Characteristics and Comorbidities With Odds of Colon or Rectal Cancer Diagnosis Through Emergency Presentation^a^

Characteristic	Colon cancer		Rectal cancer	
	AOR (95% CI)^b^	*P* value	AOR (95% CI)^b^	*P* value
Sex				
Female	0.86 (0.78-0.95)	.002	0.58 (0.32-1.04)	.07
Male	1 [Reference]	NA	1 [Reference]	NA
Age, y				
<50	2.92 (2.32-3.67)	<.001	1.35 (0.46-3.97)	.59
50-59	1.21 (1.00-1.47)	.049	1.04 (0.42-2.58)	.94
60-69	1 [Reference]	NA	1 [Reference]	NA
70-79	1.47 (1.29-1.69)	<.001	0.58 (0.24-1.41)	.23
≥80	2.06 (1.79-2.37)	<.001	1.68 (0.77-3.66)	.19
Deprivation index quintile^c^				
1	1 [Reference]	NA	1 [Reference]	NA
2	1.02 (0.88-1.18)	.82	0.86 (0.40-1.83)	.69
3	1.15 (1.00-1.34)	.06	0.56 (0.24-1.33)	.19
4	1.22 (1.06-1.40)	.007	0.69 (0.30-1.58)	.38
5	1.36 (1.19-1.54)	<.001	0.48 (0.21-1.07)	.07
Marital status				
Married	1 [Reference]	NA	1 [Reference]	NA
Single	1.28 (1.10-1.49)	.001	1.85 (0.84-4.08)	.13
Widowed	1.35 (1.20-1.53)	<.001	1.81 (0.85-3.86)	.12
Divorced	1.13 (0.88-1.45)	.36	1.85 (0.55-6.25)	.33
Specific comorbidities				
None	1 [Reference]	NA	1 [Reference]	NA
CVD	1.26 (1.13-1.41)	<.001	0.62 (0.26-1.46)	.27
Diabetes	1.11 (0.98-1.24)	.10	0.58 (0.22-1.50)	.26
Neurological diseases	1.67 (1.33-2.09)	<.001	NA	NA
Genitourinary diseases	1.07 (0.85-1.34)	.58	0.68 (0.09-5.10)	.70
Cerebrovascular diseases	1.50 (1.23-1.82)	<.001	1.39 (0.32-6.10)	.66

Abbreviations: AOR, adjusted odds ratio; CVD, cardiovascular disease; NA, not applicable.

^a^ Multivariable logistic regression (model A).

^b^ Adjusted for sex, age, deprivation index, marital status, and specific comorbidities.

^c^ Quintile 1 indicates least deprived.

**Figure 2.**
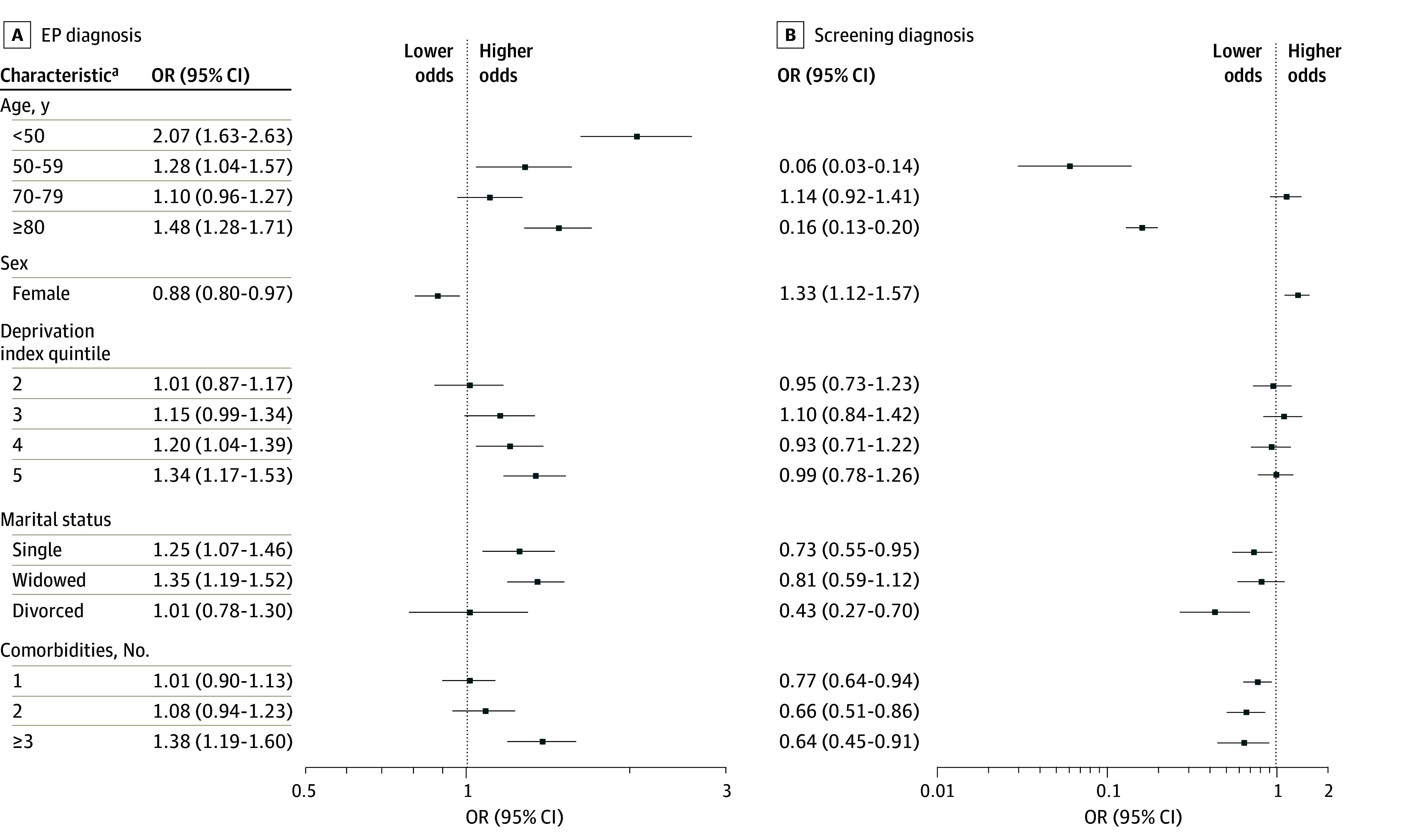
Association of Patient Characteristics and Comorbidity Count With Diagnostic Route for Colorectal Cancer Reference groups were age 60 to 69 years, male sex, deprivation index quintile 1 (indicates least deprived), married status, and no comorbidities. EP indicates emergency presentation; OR, odds ratio.

Multinomial regression revealed higher odds of the emergency presentation route for colon cancer vs the inpatient or outpatient route for patients younger than 50 years or 80 years or older, those in the highest vs lowest deprivation index quintile (AOR, 1.34; 95% CI, 1.17-1.53), those who were single or widowed (vs married), and those with the highest number of comorbidities (≥3) vs no comorbidities (AOR, 1.38; 95% CI, 1.19-1.60) (model D). Conversely, the odds of screening-detected colon cancer were significantly lower for single or divorced individuals and for those with comorbidities (1 comorbidity: AOR, 0.77 [95% CI, 0.64-0.94]; 2 comorbidities: AOR, 0.66 [95% CI, 0.51-0.86]; ≥3 comorbidities: AOR, 0.64 [95% CI, 0.45-0.91]) (Figure 2). For rectal cancer, individuals aged 80 years or older, those in deprivation index quintile 3 or higher, widowed individuals, and those with 3 or more comorbidities (AOR, 1.59; 95% CI, 1.20-2.12) had higher odds of emergency presentation diagnosis (eTable 5 in Supplement 1).

### Short-Term Mortality

Among patients with colon cancer, 615 deaths occurred within 30 days from diagnosis (390 [63.4%] in the emergency presentation route and 1 [0.2%] in the screening route); among patients with rectal cancer, 133 deaths occurred within 30 days (79 [59.4%] in the emergency presentation route and none in the screening route). Diagnosis through emergency presentation was associated with significantly higher mortality at 30 days (AOR, 4.84; 95% CI, 2.81-8.33) and 1 year (AOR, 2.77; 95% CI, 2.17- 3.53) compared with diagnosis through the inpatient or outpatient route for both colon and rectal cancer (model E) (Table 3). An increasing number of chronic conditions was associated with higher mortality at 30 days and especially at 1 year (colon cancer and ≥3 comorbidities: AOR, 2.35 [95% CI, 1.95-2.82]; rectal cancer and ≥3 comorbidities: AOR, 2.29 [95% CI 1.60-3.28]) independently of patient characteristics and cancer stage at diagnosis.

**Table 3.** Association of Patient Characteristics With Odds of Short-Term Mortality in Colon and Rectal Cancer^a^

Characteristic	Colon cancer				Rectal cancer			
	30-d mortality, AOR (95% CI)^b^	*P* value	1-y mortality, AOR (95% CI)^b^	*P* value	30-d mortality, AOR (95% CI)^b^	*P* value	1-y mortality, AOR (95% CI)^b^	*P* value
Diagnostic pathway								
IP or OP visit	1 [Reference]	NA	1 [Reference]	NA	1 [Reference]	NA	1 [Reference]	NA
Screening	NA	NA	0.43 (0.29-0.63)	<.001	NA	NA	0.25 (0.10-0.61)	.003
EP	3.09 (2.47-3.87)	<.001	2.03 (1.80-2.29)	<.001	4.84 (2.81-8.33)	<.001	2.77 (2.17-3.53)	<.001
Sex								
Female	0.85 (0.67-1.01)	.17	0.81 (0.71-0.92)	.001	1.08 (0.62-1.88)	.79	0.98 (0.77-1.25)	.87
Male	1 [Reference]	NA	1 [Reference]	NA	1 [Reference]	NA	1 [Reference]	NA
Age, y								
<50	0.45 (0.17-1.19)	.11	0.52 (0.34-0.77)	.001	0.52 (0.06-4.38)	.55	0.70 (0.35-1.40)	.31
50-59	0.91 (0.48-1.75)	.78	0.78 (0.58-1.04)	.09	0.74 (0.19-2.95)	.67	0.83 (0.50-1.35)	.45
60-69	1 [Reference]	NA	1 [Reference]	NA	1 [Reference]	NA	1 [Reference]	NA
70-79	1.47 (0.97-2.24)	0.07	1.37 (1.14-1.65)	.001	1.14 (0.45-2.93)	.78	1.63 (1.15-2.31)	.006
≥80	3.48 (2.32-5.21)	<.001	2.67 (2.20-3.24)	<.001	3.83 (1.59-9.21)	.003	3.85 (2.67-5.57)	<.001
Deprivation index quintile^c^								
1	1 [Reference]	NA	1 [Reference]	NA	1 [Reference]	NA	1 [Reference]	NA
2	0.78 (0.55-1.11)	.17	0.81 (0.67-0.98)	.03	1.08 (0.47-2.49)	.86	1.01 (0.70-1.47)	.94
3	0.88 (0.62-1.25)	.48	1.00 (0.83-1.22)	.98	1.27 (0.56-2.90)	.57	1.29 (0.89-1.85)	.18
4	1.10 (0.79-1.52)	.58	1.00 (0.83-1.20)	.98	1.90 (0.38-2.13)	.81	0.84 (0.57-1.24)	.34
5	0.80 (0.59-1.10)	.17	1.00 (0.84-1.18)	.99	1.00 (0.46-2.17)	.99	1.08 (0.77-1.51)	.66
Comorbidities, No.								
0	1 [Reference]	NA	1 [Reference]	NA	1 [Reference]	NA	1 [Reference]	NA
1	0.99 (0.74-1.34)	.96	1.15 (0.99-1.35)	.07	0.93 (0.45-1.91)	.84	1.10 (0.82-1.47)	.52
2	1.46 (1.07-1.98)	.02	1.72 (1.45-2.04)	<.001	1.38 (0.63-3.01)	.42	1.16 (0.82-1.65)	.40
≥3	1.89 (1.38-2.59)	<.001	2.35 (1.95-2.82)	<.001	1.81 (0.84-3.88)	.13	2.29 (1.60-3.28)	<.001
Cancer stage at diagnosis								
1	1 [Reference]	NA	1 [Reference]	NA	1 [Reference]	NA	1 [Reference]	NA
2	1.89 (1.08-3.29)	.03	1.39 (1.06-1.81)	.02	1.44 (0.26-8.03)	.68	2.18 (1.18-4.00)	.01
≥3	5.16 (3.09-8.64)	<.001	7.44 (5.85-9.46)	<.001	7.48 (1.79-31.15)	.006	9.51 (5.61-16.11)	<.001
Marital status								
Married	1 [Reference]	NA	1 [Reference]	NA	1 [Reference]	NA	1 [Reference]	NA
Single	1.62 (1.12-2.34)	.01	1.43 (1.16-1.75)	<.001	1.69 (0.74-3.88)	.22	1.35 (0.92-1.97)	.13
Widowed	1.26 (0.96-1.66)	.09	1.42 (1.22-1.66)	<.001	1.36 (0.71-2.57)	.35	1.37 (1.02-1.86)	.04
Divorced	1.26 (0.66-2.38)	.49	1.08 (0.77-1.51)	.67	1.02 (0.22-4.60)	.98	1.32 (0.74-2.36)	.34

Abbreviations: EP, emergency presentation; IP, inpatient; NA, not applicable; OP, outpatient.

^a^ Multivariable logistic regression (model E).

^b^ Adjusted for diagnostic pathway, socioeconomic characteristics, comorbidity count, and cancer stage at diagnosis.

^c^ Quintile 1 indicates least deprived.

### CRC Diagnostic Route Before vs During the COVID-19 Pandemic

During the first year of the COVID-19 pandemic (March to December 2020), 1392 cases of CRC were recorded within the ATS of Milan. The COVID-19 lockdown period was associated with higher odds of emergency diagnoses compared with the prepandemic period (AOR, 1.32; 95% CI, 1.15-1.52), but there was no difference in odds of screening diagnoses (AOR, 0.85; 95% CI, 0.63-1.14) (eTable 6 in Supplement 1).

## Discussion

Using cancer registry and administrative health data, we analyzed diagnostic routes and outcomes of CRC, accounting for patient socioeconomic characteristics and comorbidities. Emergency presentation diagnoses were frequent, occurring in 35.6% of colon cancer cases and 22.6% of rectal cancer cases. Approximately half of the patients (44.6% with colon cancer and 57.6% with rectal cancer) had 1 or more preexisting chronic conditions, the presence of which were associated with a higher likelihood of emergency presentation diagnosis for both colon and rectal cancer and a lower likelihood of screening-detected colon cancer. Emergency presentation diagnoses were more common among the youngest and oldest patients, those with lower socioeconomic status, widowed individuals, and those with advanced-stage cancer. More patients diagnosed during emergency presentation were at advanced stages, while more of those diagnosed through screening had early stages. Emergency presentation diagnosis was associated with higher short-term mortality. During the first year of the COVID-19 pandemic, the odds of emergency presentation diagnosis were higher compared with the prepandemic period.

To our knowledge, this is the first population-based study on routes of cancer diagnosis among patients in Italy and is one of the few such studies available for Central and Southern Europe.^28,29^ A Spanish study^30^ reported a higher percentage of emergency presentation diagnoses than other studies^31,32,33,34^ (around 40%). This increase was attributed to general practitioners referring patients with suspected cancer to emergency presentation due to limited access to diagnostic investigations and outpatient appointments. Our findings are in line with studies from Northern Europe, the UK, and North America, reporting between 17% and 26% of CRC diagnoses through emergency presentation.^31,32,33^ In a Swedish study,^34^ the proportion of CRC cases diagnosed through emergency presentation was slightly lower (21.5%). On the contrary, a study^35^ from the Norwegian Cancer Registry reported a higher proportion of diagnoses through emergency presentation (35% for colon and 15% for rectal cancer). Overall, the proportion of emergency presentation CRC diagnosis has been shown to be high, even in countries known for their high standards of care and favorable cancer outcomes. Despite well-established screening programs and high-quality medical infrastructure, there are persistent gaps in early detection and timely intervention. These findings underscore the need for enhanced efforts to strengthen early detection strategies and boost participation in screening programs, particularly among high-risk populations and individuals with comorbidities.

Across countries, emergency presentation diagnoses occur less often for rectal cancers, as these cancers more frequently present with rectal bleeding, an easily recognizable alarm symptom urging investigations. In contrast, colon cancers often present with changes in bowel habits or anemia, which have been previously associated with a longer time before patients seek help from their doctor, with delays after symptomatic presentation, and with missed opportunities for early diagnosis.^36^

To our knowledge, the current study is one of the first in Italy to investigate the association of comorbidity burden and specific comorbidities with diagnostic routes for patients with CRC. As chronic diseases and cancer incidence increase with age, a substantial proportion of patients diagnosed with cancer have preexisting comorbidities. This trend will likely continue as the population ages and medical treatments advance.^37^ Our study demonstrated that emergency presentation diagnoses were significantly associated with certain chronic conditions and with having 3 or more comorbidities. In line with our findings, conditions like dementia (OR, 2.46; 95% CI, 2.18-2.79) and congestive heart failure (OR, 1.49; 95% CI, 1.37-1.61)^33^ have already been associated with higher risks of emergency presentation.^38,39^ Despite frequent health care visits, these comorbidities may delay timely cancer investigations by complicating diagnostic routes, requiring management of severe conditions before invasive tests,^40^ providing alternative explanations for symptoms, and affecting health care practitioners’ decision-making.^41,42,43^

In Italy, the target population for CRC screening includes all men and women aged 50 to 74 years. Our results showed that individuals in this age group had the lowest risk of emergency presentation diagnoses, probably due to screening campaigns. Conversely, 47.3% of patients younger than 50 years and even a higher percentage of those older than 90 years (53.4%) had an emergency presentation diagnosis. A small number of patients in our study (n = 10 [0.7%]) was screened outside the target age range, with the majority of those (7 [70.0%]) being aged 49 years, as screening invitations are based on birth year, not exact age. Our study showed an association between the number of comorbidities and a reduced likelihood of screening for colon cancer detected by screening. The odds of short-term mortality after emergency presentation were higher than after screening or inpatient or outpatient diagnosis even after adjustment for socioeconomic characteristics, cancer stage at diagnosis, and comorbidity count.^35,44,45^

This study is among the first in Italy to provide an overview of cancer diagnoses during the first year of the COVID-19 pandemic. Consistent with national reports,^46^ our findings indicated a significant increase in CRC diagnoses through emergency presentation during the pandemic.

The implications of our study findings for practice and policy include the need for targeted strategies to support CRC screening in people with chronic conditions who may face barriers in accessing screening. Interventions are also needed to reduce cancer diagnoses following emergency presentation. These might include risk assessment tools and clinical guidelines for patients with specific morbidities or multimorbidity. Improving integration between primary and secondary care might be particularly important for enabling earlier cancer diagnosis in patients with multimorbidity.

### Strengths and Limitations

Strengths of this study include the development of comprehensive methods to categorize cancer diagnosis routes using cancer registry data linked to administrative health databases. This innovative approach, based on the use of large and routinely available datasets, enhances cancer surveillance and identifies areas needing improvement, and it can be applied in other health care settings. The high-quality data from cancer registries ensure the reliability and validity of our findings, supporting the generalizability of our methods.

However, limitations of this study arose from the absence of clinical data sources. While our algorithm assumed that clinical activity up to 6 months (and focusing on the 30 days) before cancer diagnosis was related to cancer diagnosis, this may not always be the case. In Italy, individually linked electronic health records on symptoms are not available at the population level. Consequently, data on patient symptoms at the time of diagnosis are not accessible, limiting our ability to differentiate between symptomatic and asymptomatic cases. Missing information on the cancer stage at diagnosis might affect associations between stage at diagnosis and risk estimates, but stage distribution over time remained stable, with a bias in a specific direction being unlikely. This study did not evaluate causal relationships but reported observed associations. While another study^47^ examined the role of emergency presentation in mediating the association of comorbidities with survival, further research using causal inference methods is needed to disentangle the interplay of diagnostic route, comorbidities, socioeconomic factors, and survival. While methods such as time-to-event analysis could be used, our approach examining short-term mortality was chosen to ensure comparability of our results with national and international studies^48,49^ on routes to diagnosis and diagnostic delays.^1^ Short-term mortality is considered a possible indicator of diagnostic delay, as it is associated with more advanced cancer stages at diagnosis. Additionally, the study data cover only the years 2014 to 2020, posing a temporal limitation; further studies are also needed to assess the association during the entire pandemic period.

## Conclusions

In this population-based cohort study in Italy, emergency diagnosis occurred in more than 1 in 3 patients with colon cancer. Comorbidities were associated with a lower likelihood of screening detection, higher risk of emergency diagnosis, and higher mortality. Tailored interventions are needed to facilitate screening, to reduce emergency cancer diagnoses, and to improve outcomes for a large number of patients with chronic conditions. Understanding differences in the likelihood of emergency presentation cancer diagnoses across countries can help identify approaches for improving the timely diagnosis of cancer and cancer outcomes. The routine collection and analysis of electronic health data hold relevant potential for conducting these analyses. This ongoing research is essential to adapt and optimize cancer care amid evolving health care challenges, particularly with aging populations and increasing multimorbidity.

## Notes

Supplement 1. **eFigure.** Directed Acyclic Graph Illustrating Associations Between Variables in the Analysis **eTable 1.** Thirty-Day and 6-Month Colorectal Cancer Diagnostic Routes **eTable 2.** Stage at Cancer Diagnosis and Mortality by Diagnostic Route **eTable 3.** Multivariable Logistic Regression Assessing the Association Between Patient Characteristics, Number of Comorbidities, and Emergency Cancer Diagnosis **eTable 4.** Multivariable Logistic Regression Assessing the Association Between Patient Characteristics, Stage at Diagnosis, Number of Comorbidities, and Emergency Cancer Diagnosis **eTable 5.** Multinomial Logistic Regression Analysis Assessing the Association Between Patient Characteristics, Comorbidity Count, and Diagnostic Route for Rectal Cancer **eTable 6.** Multinomial Logistic Regression Analysis Comparing the First Year of COVID-19 With the Prepandemic Period for Colon Cancer

Supplement 2.
Data Sharing Statement
