# Supplementary material for: Comorbidities, Socioeconomic Status, and Colorectal Cancer Diagnostic Route
Source: JAMA Netw Open. 2025 May 6;8(5):e258867. doi: 10.1001/jamanetworkopen.2025.8867 (PMC12056571; doi:10.1001/jamanetworkopen.2025.8867)
Supplement: Supplement 1. — eFigure. Directed Acyclic Graph Illustrating Associations Between Variables in the Analysis eTable 1. Thirty-Day and 6-Month Colorectal Cancer Diagnostic Routes eTable 2. Stage at Cancer Diagnosis and Mortality by Diagnostic Route eTable 3. Multivariable Logistic Regression Assessing the Association Between Patient Characteristics, Number of Comorbidities, and Emergency Cancer Diagnosis eTable 4. Multivariable Logistic Regression Assessing the Association Between Patient Characteristics, Stage at Diagnosis, Number of Comorbidities, and Emergency Cancer Diagnosis eTable 5. Multinomial Logistic Regression Analysis Assessing the Association Between Patient Characteristics, Comorbidity Count, and Diagnostic Route for Rectal Cancer eTable 6. Multinomial Logistic Regression Analysis Comparing the First Year of COVID-19 With the Prepandemic Period for Colon Cancer [file jamanetwopen-e258867-s001.pdf]

Supplementary Online Content

Pennisi F, Buzzoni C, Russo AG, Gervasi F, Braga M, Renzi C. Comorbidities, socioeconomic status, and colorectal cancer diagnostic route. *JAMA Netw Open*. 2025;8(5):e258867. doi:10.1001/jamanetworkopen.2025.8867

eFigure. Directed Acyclic Graph Illustrating Associations Between Variables in the Analysis

eTable 1. Thirty-Day and 6-Month Colorectal Cancer Diagnostic Routes

eTable 2. Stage at Cancer Diagnosis and Mortality by Diagnostic Route

eTable 3. Multivariable Logistic Regression Assessing the Association Between Patient Characteristics, Number of Comorbidities, and Emergency Cancer Diagnosis

eTable 4. Multivariable Logistic Regression Assessing the Association Between Patient Characteristics, Stage at Diagnosis, Number of Comorbidities, and Emergency Cancer Diagnosis

eTable 5. Multinomial Logistic Regression Analysis Assessing the Association Between Patient Characteristics, Comorbidity Count, and Diagnostic Route for Rectal Cancer

eTable 6. Multinomial Logistic Regression Analysis Comparing the First Year of COVID-19 With the Prepandemic Period for Colon Cancer

This supplementary material has been provided by the authors to give readers additional information about their work.

© 2025 Pennisi F et al. *JAMA Network Open*.

eFigure. Directed Acyclic Graph (DAG) illustrating the associations between the variables in the analysis.

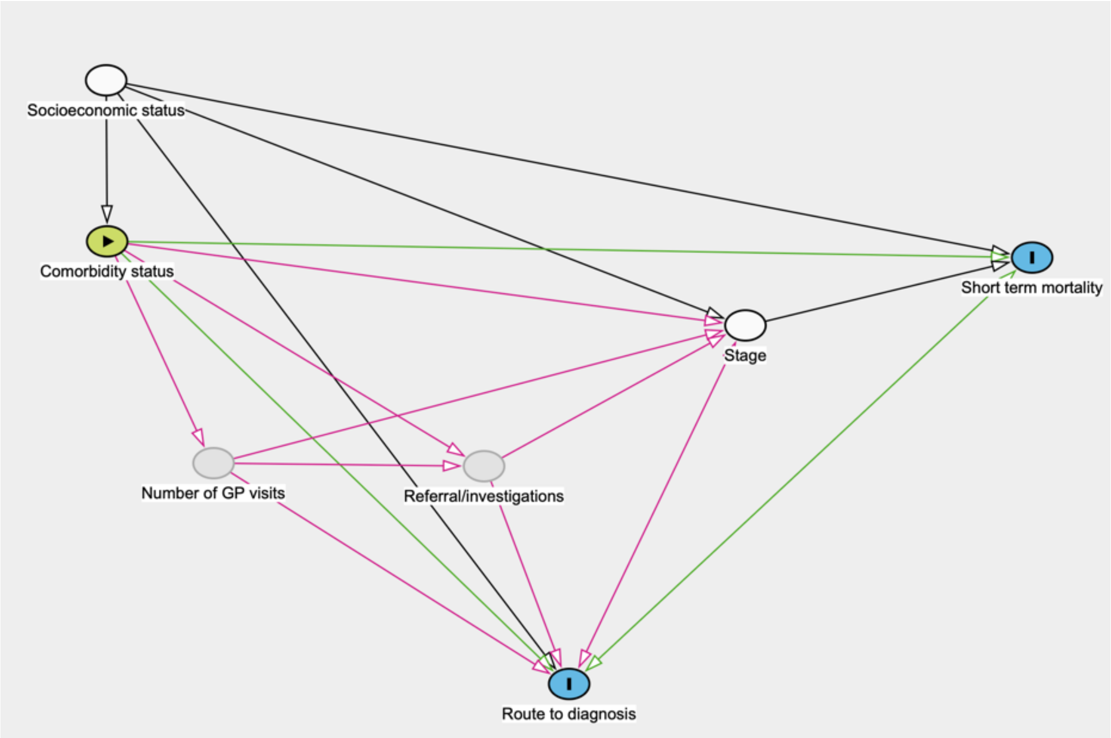

Green arrows: causal paths
Red arrows: biasing paths
Blue circles: outcome variables
Green circle: exposure variable
White circles: adjusted variables
Grey circles: unmeasured variables

© 2025 Pennisi F et al. *JAMA Network Open*.

eTable 1. 30 days and 6 months colorectal cancer diagnostic route.

COLON				RECTUM		
Period before diagnosis (% col)						
	30 days	6 months	**Total**	30 days	6 months	**Total**
SCREENING						
	585	296	881	237	109	346
	6.1%	30.3%	8.4%	7.2%	34.0%	9.5%
EP						
	3.553	185	3.738	763	60	823
	37.3%	19.0%	35.6%	23.0%	18.7%	22.6%
IP/OP						
	5400	495	5895	2314	152	2466
	56.6%	50.7%	56.1%	69.8%	47.4%	67.8%
TOTAL						
	9538	976	10514	3.314	321	3635
	90.7%	9.3%	100.00%	91.2%	8.8%	100.00%

EP: emergency presentation; IP: inpatient; OP: outpatient

© 2025 Pennisi F et al. *JAMA Network Open*.

eTable 2. Stage at cancer diagnosis and mortality by diagnostic routes.

	COLON					RECTUM				
	Total	EP	Screening	IP/OP	P value	Total	EP	Screening	IP/OP	P value
	N= 10514	N=3738 (35.6%)	N=881 (8.4%)	N=5895 (56.1%)		N=3635	N=823 (22.6%)	N=347 (9.5%)	N=2466 (67.8%)	
Stage at diagnosis										
1	1609	328	272	1.009		604	82	111	411	
	16.5%	9.5%	32.4%	18.4%		18.5%	11.7%	34.8%	18.4%	
2	2804	1.037	201	1.566		604	127	47	430	
	28.7%	30.0%	24.0%	28.6%		18.5%	18.1%	14.7%	19.2%	
3	3487	1222	291	1974	<0.001	1481	308	144	1029	<0.001
	35.7%	35.4%	34.7%	36.1%		45.4%	43.8%	45.1%	45.9%	
4	1865	866	75	924		573	186	17	370	
	19.1%	25.1%	8.9%	16.9%		17.6%	26.5%	5.3%	16.5%	
Missing	750					373				
	7.1%					10.3%				
Mortality										
<30 days	615	390	1	224		133	79	0	54	
	5.9%	10.4%	0.1%	3.8%		3.7%	9.6%	0.0%	2.2%	
>30 days and <6 months	1114	562	16	536	<0.001	324	151	3	170	<0.001
	10.6%	15.0%	1.8%	9.1%		8.9%	18.4%	0.9%	6.9%	
>6 months and <1 year	612	272	20	320		191	71	4	116	
	5.8%	7.3%	2.3%	5.4%		5.3%	8.6%	1.2%	4.7%	

EP: emergency presentation; IP: inpatient; OP: outpatient

© 2025 Pennisi F et al. *JAMA Network Open*.

eTable 3. Multivariable logistic regression assessing the association between patient characteristics, number of comorbidities and emergency cancer diagnosis (Model B).

COLON Adjusted			RECTUM Adjusted	
	EP (EP=1) (adjusted) OR (95% IC)	P value	EP (EP=1) (adjusted) OR (95% IC)	P value
Sex (Ref M)				
F	0.83 (0.75-0.91)	0.14	0.61 (0.34-1.10)	0.10
Age (Ref. 60-69)				
<50	3.00 (2.39-3.76)	<0.001	1.15 (0.39-3.43)	0.80
50-59	1.24 (1.02-1.50)	0.03	0.90 (0.36-2.25)	0.82
70-79	1.41 (1.23-1.61)	<0.001	0.74 (0.31-1.75)	0.49
>=80	1.98 (1.72-2.28)	<0.001	2.00 (0.92-4.39)	0.08
Marital status (Ref.Married)				
Single	1.32 (1.14-1.53)	<0.001	1.70 (0.77-3.76)	0.19
Widowed	1.34 (1.19-1.51)	<0.001	2.09 (0.98-4.42)	0.06
Divorced	1.16 (0.90-1.48)	0.26	1.81 (0.53-6.13)	0.34
Educational level (Ref. None/primary school)				
Secondary school	0.90 (0.80-1.01)	0.07	1.56 (0.66-3.73)	0.32
Diploma/degree/PhD	0.72 (0.64-0.81)	<0.001	2.36 (1.03-5.38)	0.04
Comorbidity count (Ref 0)				
1	1.12 (1.01-1.25)	0.03	0.47 (0.23-0.99)	0.05
2	1.27 (1.10-1.46)	<0.001	0.27 (0.06-1.13)	0.07
3+	1.78 (1.47-2.16)	<0.001	0.30 (0.04-2.25)	0.24

EP: emergency presentation

© 2025 Pennisi F et al. *JAMA Network Open*.

eTable 4. Multivariable logistic regression assessing the association between patient characteristics, stage at diagnosis, number of comorbidities and emergency cancer diagnosis (Model C).

	COLON Adjusted		RECTUM Adjusted	
	EP (EP=1) (adjusted) OR (95% IC)	P value	EP (EP=1) (adjusted) OR (95% IC)	P value
Sex (Ref M)				
F	0.85 (0.77-0.94)	0.002	0.53 (0.26-1.10)	0.09
Age (Ref. 60-69)				
<50	2.84 (2.24-3.61)	<0.001	0.61 (1.13-2.88)	0.53
50-59	1.23 (1.00-1.49)	0.04	0.88 (0.32-2.48)	0.81
70-79	1.49 (1.29-1.71)	<0.001	0.59 (0.22-1.59)	0.30
>=80	2.06 (1.78-2.38)	<0.001	1.73 (0.71-4.19)	0.23
Deprivation index (Ref. 1)				
2	1.02 (0.88-1.18)	0.83	0.91 (0.35-2.33)	0.84
3	1.15 (0.98-1.34)	0.08	0.66 (0.24-1.87)	0.44
4	1.20 (1.03-1.38)	0.02	0.72 (0.26-2.00)	0.52
5	1.36 (1.19-1.56)	<0.001	0.62 (0.24-1.59)	0.32
Marital status (Ref. Married)				
Single	1.28 (1.10-1.50)	0.001	1.91 (0.74-4.96)	0.18
Widowed	1.34 (1.18-1.52)	<0.001	1.13 (0.42-3.08)	0.80
Divorced	1.18 (0.91-1.52)	0.21	2.52 (0.73-8.71)	0.15
Comorbidity count (Ref 0)				
1	1.14 (1.02-1.26)	0.02	0.65 (0.29-1.47)	0.30
2	1.28 (1.11-1.49)	0.001	0.20 (0.03-1.48)	0.12
3+	1.82 (1.48-2.24)	<0.001	0.51 (0.07-3.89)	0.52
Stage at diagnosis (Ref 1)				
2	2.15 (1.84-2.53)	<0.001	0.75 (0.28-2.06)	0.58
3+	2.41 (2.08-2.79)	<0.001	0.78 (0.35-1.71)	0.53

EP: emergency presentation

© 2025 Pennisi F et al. *JAMA Network Open*.

eTable 5. Multinomial logistic regression analysis assessing the association between patient characteristics, comorbidity count and the diagnostic route for rectal cancer (Model D).

RECTUM *(Ref. =IP/OP)*	**ER (adjusted) OR (95% IC)**	**P value**	**Screening (adjusted) OR (95% IC)**	**P value**
Age (Ref 60-69)				
<50	0.76 (0.47-1.23)	0.26	0.06 (0.02-0.20)	<0.001
50-59	1.03 (0.72-1.46)	0.87	1.13 (0.83-1.54)	0.43
70-79	1.22 (0.94-1.59)	0.14	0.19 (0.13-0.27)	<0.001
>=80	2.03 (1.54-2.67)	<0.001	NA	NA
Sex (Ref Male)				
Female	1.07 (0.89-1.30)	0.44	1.69 (1.30-2.20)	<0.001
Deprivation index (Ref. 1)				
2	1.04 (0.77-1.40)	0.81	1.79 (1.17-2.72)	0.007
3	1.36 (1.02-1.82)	0.04	1.82 (1.19-2.80)	0.006
4	1.56 (1.17-2.08)	0.002	1.34 (0.85-2.12)	0.20
5	1.37 (1.06-1.79)	0.02	1.31 (0.87-1.99)	0.20
Marital status (Ref. Married)				
Single	1.08 (0.80-1.46)	0.61	0.60 (0.39-0.92)	0.02
Widowed	1.33 (1.05-1.70)	0.02	0.53 (0.31-0.94)	0.03
Divorced	1.43 (0.92-2.20)	0.11	0.90 (0.49-1.66)	0.73
Comorbidity count (Ref 0)				
1	0.99 (0.79-1.24)	0.95	1.23 (0.91-1.66)	0.18
2	1.20 (0.92-1.56)	0.18	1.07 (0.70-1.64)	0.75
3+	1.59 (1.20-2.12)	0.002	1.20 (0.70-2.05)	0.52

EP: emergency presentation; IP: inpatient; OP: outpatient

© 2025 Pennisi F et al. *JAMA Network Open*.

eTable 6. Multinomial logistic regression analysis comparing the first year (March-December 2020) of COVID-19 with the pre-pandemic period (2014-February 2020) for colon cancer.

**COLON-RECTUM 2020**(Ref.=IP/OP)	**EP (adjusted) OR (95% IC)**	**P value**	**Screening (adjusted) OR (95% IC)**	**P value**
Pandemic period (Ref. Pre-pandemic period)				
Pandemic period	1.32 (1.15-1.52)	<0.001	0.85 (0.63-1.14)	0.27
Age (Ref 60-69)				
<50	1.85 (1.48-2.30)	<0.001	0.05 (0.02-0.12)	<0.001
50-59	1.25 (1.03-1.51)	0.02	1.14 (0.93-1.40)	0.19
70-79	1.07 (0.94-1.22)	0.34	0.18 (0.15-0.22)	<0.001
>=80	1.43 (1.25-1.63)	<0.001	0.001 (<0.001-0.009)	<0.001
Sex (Ref Male)				
Female	0.87 (0.79-0.95)	0.002	1.29 (1.10-1.51)	0.002
Deprivation index (Ref. 1)				
2	1.07 (0.94-1.23)	0.32	0.99 (0.77-1.27)	0.92
3	1.14 (0.99-1.31)	0.07	1.06 (0.83-1.36)	0.64
4	1.21 (1.05-1.38)	0.006	1.00 (0.78-1.29)	0.98
5	1.35 (1.20-1.53)	<0.001	1.02 (0.81-1.29)	0.85
Marital status (Ref. Married)				
Single	1.29 (1.12-1.50)	<0.001	0.70 (0.54-0.91)	0.008
Widowed	1.37 (1.22-1.53)	<0.001	0.74 (0.55-1.01)	0.05
Divorced	1.02 (0.80-1.29)	0.90	0.46 (0.29-0.71)	<0.001
Comorbidity count (Ref 0)				
1	1.03 (0.94-1.14)	0.51	0.63 (0.52-0.77)	<0.001
2	1.13 (0.99-1.29)	0.07	0.64 (0.47-0.87)	0.004
3+	1.65 (1.38-1.98)	<0.001	0.53 (0.30-0.91)	0.022

EP: emergency presentation; IP: inpatient; OP: outpatient

© 2025 Pennisi F et al. *JAMA Network Open*.
