# Supplementary material for: Comorbidities, Socioeconomic Status, and Colorectal Cancer Diagnostic Route
Source: JAMA Netw Open. 2025 May 6;8(5):e258867. doi: 10.1001/jamanetworkopen.2025.8867 (PMC12056571; doi:10.1001/jamanetworkopen.2025.8867)
Supplement: Supplement 2. — Data Sharing Statement [file jamanetwopen-e258867-s002.pdf]

Data Sharing Statement

Pennisi. Comorbidities, Socioeconomic Status, and Colorectal Cancer Diagnostic Route. *JAMA Netw Open*. Published May 06, 2025. doi:10.1001/jamanetworkopen.2025.8867

Data
Data available: No

Additional Information
Explanation for why data not available: The data supporting the findings of this article are available at an aggregated level from the authors upon reasonable request and with permission of ATS of Milan. Requests to access should be directed to Carlotta Buzzoni, Scientific Manager of the study, email: cbuzzoni@ats-milano.it
